# Comparing ultrastable lasers at 7 × 10^−17^ fractional frequency instability through a 2220 km optical fibre network

**DOI:** 10.1038/s41467-021-27884-3

**Published:** 2022-01-11

**Authors:** M. Schioppo, J. Kronjäger, A. Silva, R. Ilieva, J. W. Paterson, C. F. A. Baynham, W. Bowden, I. R. Hill, R. Hobson, A. Vianello, M. Dovale-Álvarez, R. A. Williams, G. Marra, H. S. Margolis, A. Amy-Klein, O. Lopez, E. Cantin, H. Álvarez-Martínez, R. Le Targat, P. E. Pottie, N. Quintin, T. Legero, S. Häfner, U. Sterr, R. Schwarz, S. Dörscher, C. Lisdat, S. Koke, A. Kuhl, T. Waterholter, E. Benkler, G. Grosche

**Affiliations:** 1 National Physical Laboratory (NPL), Teddington, TW11 0LW UK; 2 Laboratoire de Physique des Lasers (LPL), Université Paris 13, CNRS, Villetaneuse, France; 3 LNE-SYRTE, Observatoire de Paris - Université PSL, CNRS, Sorbonne Université, LNE, Paris, France; 4 Real Instituto y Observatorio de la Armada (ROA), 11100 San Fernando, Cádiz Spain; 5 RENATER, Paris, France; 6 Physikalisch-Technische Bundesanstalt (PTB), Bundesallee 100, 38116 Braunschweig, Germany

**Keywords:** Optical sensors, Fluorescence spectroscopy, Optical metrology, Seismology, Fibre optics and optical communications

## Abstract

Ultrastable lasers are essential tools in optical frequency metrology enabling unprecedented measurement precision that impacts on fields such as atomic timekeeping, tests of fundamental physics, and geodesy. To characterise an ultrastable laser it needs to be compared with a laser of similar performance, but a suitable system may not be available locally. Here, we report a comparison of two geographically separated lasers, over the longest ever reported metrological optical fibre link network, measuring 2220 km in length, at a state-of-the-art fractional-frequency instability of 7 × 10^−17^ for averaging times between 30 s and 200 s. The measurements also allow the short-term instability of the complete optical fibre link network to be directly observed without using a loop-back fibre. Based on the characterisation of the noise in the lasers and optical fibre link network over different timescales, we investigate the potential for disseminating ultrastable light to improve the performance of remote optical clocks.

## Abstract (web summary)

Precision measurement plays an important role in frequency metrology and optical communications. Here the authors compare two geographically separate ultrastable lasers at 7 × 10^−17 ^fractional frequency instability over a 2220 km optical fibre link and these measurements can be useful for dissemination of ultrastable light to distant optical clocks.

## Introduction

Time and frequency standards operating in the optical domain are demonstrating unprecedented measurement precision^1–7^. Their high frequency stability, defined as their ability to keep their frequency over time with limited fluctuations, makes them powerful measurement tools to test the fundamental laws of nature^8–13^ and sensitive probes for applications such as relativistic geodesy^14–16^. Ultrastable lasers are at the core of the measurement capability of optical time and frequency standards as they basically set their precision performance. Modern ultrastable lasers are so stable that their performance can only be assessed by comparing similar systems^17,18^. Alternative methods to measure laser noise require a state-of-the-art optical lattice clock available locally^19,20^. Unfortunately, state-of-the-art lasers and optical lattice clocks are not widely accessible yet due to their complexity and cost, therefore a system for comparison may not be easily available in near proximity. This can make it difficult to evaluate and optimise the performance of ultrastable lasers and can prevent applications that critically depend on stable optical references, such as optical atomic clocks, from realising their full measurement potential.

Here, we show that it is possible to compare two geographically distant ultrastable lasers^21,22^, with state-of-the-art fractional frequency instability^17,18^ below 1 × 10^−16^, through the longest ever reported metrological optical fibre link network^21–30^, at averaging times that are relevant for applications such as optical clocks.

The two state-of-the art ultrastable lasers considered here also allow a direct measurement of the total end-to-end fibre link noise at averaging times <10 s, achieved without having to loop back light through a second link, as typically realised to characterise a link. Based on the measurements of the noise of the ultrastable lasers and fibre link, we investigate a strategy for disseminating ultrastable light to distant users. This technique has the potential to enable the large community of optical atomic clock users to bring the performance of their systems to the level of those having direct access to state-of-the-art ultrastable lasers, leading to significantly relaxed requirements on their local lasers.

## Results

### Comparison of ultrastable lasers through the 2220 km fibre link

**Fig. 1:**
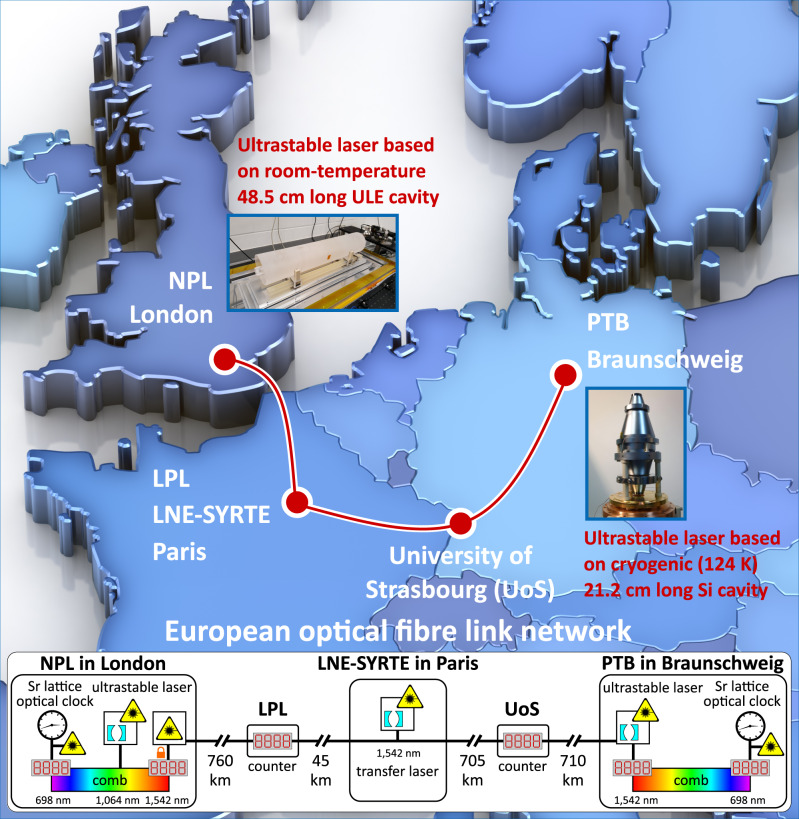
Ultrastable laser and optical fibre link network experiment layout. Schematic of the 2220 km long optical fibre link connecting NPL in London (UK), LPL and LNE-SYRTE in Paris (France), University of Strasbourg UoS (France) and PTB in Braunschweig (Germany). The link enables distant state-of-the-art ultrastable lasers at NPL and PTB to be compared. The NPL laser at 1064 nm is based on a room-temperature 48.5 cm long ultra-low-expansion (ULE) optical cavity and its stability is transferred via an optical frequency comb to a laser at 1542 nm. The PTB laser at 1542 nm is based on a 21.2 cm long silicon cavity at 124 K. The Sr optical lattice clocks at NPL and PTB are used to verify the stability performance of the ultrastable lasers. The picture of the PTB silicon cavity is taken from ref. ^58^.

The ultimate fractional frequency instability ${\sigma }_{y}$ of a laser, stabilised to an optical reference cavity, is limited by the fundamental flicker frequency noise originating from Brownian thermal-mechanical fluctuations of the cavity mirrors’ substrate and coating, according to^31,32^

1 $${\sigma }_{y}\propto \frac{1}{{L}_{{{{{{\rm{cav}}}}}}}}\sqrt{\frac{{{{k}}}\,{{{T}}}}{E}}$$

where ${L}_{{{{{{\rm{cav}}}}}}}$ is the spacer length, *k* the Boltzmann constant, and *T* and *E* the absolute temperature and mechanical Young’s modulus of the mirror substrates, respectively. The two lasers used in this work (Fig. 1) employ different strategies to minimise the thermal noise: increasing ${L}_{{{{{{\rm{cav}}}}}}}$ and *E*, and reducing *T*. One, developed at NPL^33^, is based on a 48.5 cm long optical cavity made of ultra low expansion (ULE) glass spacer and fused silica (*E*_FS_ = 73.1 GPa) mirrors, operated at room-temperature and with an estimated thermal noise floor at $6 \,\times {10}^{-17}$ fractional frequency. The other system, at PTB^18^, uses a 21.2 cm long optical cavity made of crystalline silicon (*E*_Si_ = 187.5 GPa) spacer and mirrors, operating at 124 K and with a measured thermal noise floor at $4\times {10}^{-17}$.

**Fig. 2:**
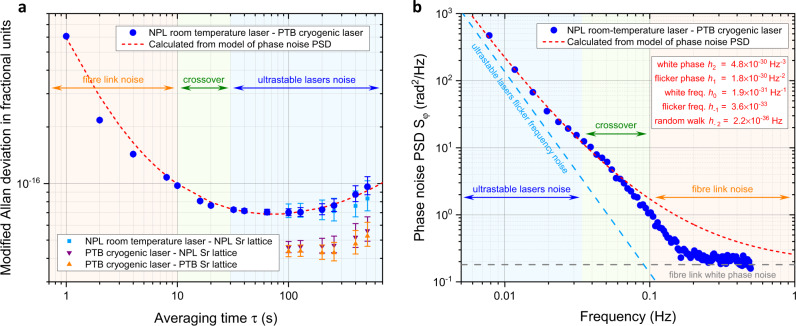
Experimental results of the comparison of ultrastable lasers through the optical fibre link. **a** Combined instability measured comparing the NPL room-temperature laser and PTB cryogenic laser (blue circles) through the 2220 km long European fibre link network. A linear drift of 40 mHz s^−1^ has been removed in the instability evaluation. NPL laser instability evaluated by the NPL strontium lattice clock (cyan squares). PTB laser instability measured by the PTB (orange upwards triangles) and NPL (magenta downwards triangles) strontium lattice clocks. Calculated instability for a simplified model for the phase noise power spectral density (PSD) (red dashed line). The orange, green and blue shadows highlight the main contributors for the different ranges of integration time, respectively, the fibre link noise below 10 s, a crossover region from 10 s to 30 s, the noise of the ultrastable lasers above 30 s. The error bars represent the $1\,\sigma$ uncertainty of the modified Allan deviation. **b** Measured ensemble phase noise of the ultrastable lasers at PTB and NPL and the fibre link (blue circles). No information about the link noise is available for frequencies above 0.5 Hz since the measurements are based on dead-time-free $\varLambda$-counted data with report interval of 1 s. The blue, green and orange shadows highlight the main contributors for the different ranges of frequencies, respectively, the ultrastable lasers noise below 30 mHz, a crossover region from 30 mHz to 100 mHz, the fibre link noise above 100 mHz.

In this work to compare the two distant lasers, each with state-of-the-art stability, we used the European metrological optical fibre link network^24,27–30^ (Fig. 1) connecting NPL to PTB via LPL, LNE-SYRTE, and the University of Strasbourg (UoS), with a total fibre link length of 2220 km, the longest ever reported metrological optical fibre link network^21–30^. In this comparison the modified Allan deviation (MDEV) of dead-time-free $\varLambda$-counted^34,35^ beat frequency measurements revealed a combined flicker frequency noise floor at $7\times {10}^{-17}$, from 30 s to 200 s (Fig. 2). We have independently verified the performance of the PTB cryogenic laser to be at the expected $4\times {10}^{-17}$ MDEV flicker frequency thermal noise floor^18^ through both local and remote measurements using the Sr lattice clocks at PTB and NPL, starting from 100 s owing to the 10–20 s attack time of the atomic servo. We deduce that the flicker frequency noise floor of the NPL ultrastable laser is at $6\times {10}^{-17}$ MDEV ($7\times {10}^{-17}$ standard Allan deviation), which is at the estimated thermal noise level for this system and at the same level as previously reported state-of-the-art stability for a room-temperature system^17,18^. Achieving this performance required the active vibration isolation to be improved as described in the Methods section. The Sr lattice clock at NPL has been used to verify the instability of the local laser for timescales longer than 100 s, leading to a measured flicker frequency floor at $7\times {10}^{-17}$ MDEV, close to that estimated by comparing the two ultrastable lasers. In Supplementary Information we provide the measured instability of the comparison of the distant ultrastable lasers for additional datasets (see Supplementary Fig. 1).

### Direct evaluation of the total link noise with ultrastable lasers

We used the comparison of distant ultrastable lasers to directly measure the short-term frequency instability of the entire measurement chain of the 2220 km fibre link network. This comparison is dominated at 1 s averaging time by delay-unsuppressed noise due to fundamental limitations of the fibre link noise cancellation^36^, where the intrinsic transit time both causes the returned signal to sample the fibre noise at a slightly later point in time, and limits the bandwidth available for noise suppression. A strategy to minimise this effect is to divide the link into smaller segments to increase the stabilisation bandwidth for each segment. Usually, frequency transfer performance is characterised by establishing one stabilised link in a loop of two fibres connected at the far end^23,25,26^ or by cascading two independently stabilised links that form a loop^24,28,30^. The latter configuration is used in the European fibre network^29^, which mainly consists of one metropolitan 45 km long fibre link and three long-haul fibre links of the similar lengths 705 km, 710 km and 760 km. The combined instability of the cascaded links A-B-A establishes an upper limit for the single link A-B. In the case of two cascaded fibres running parallel in the same cable, the fact that noise contributions are highly correlated^30^ can be exploited to derive a more stringent limit on the noise of a single link^23,26^. These loop-back measurements have been employed for the most rigorous fibre link frequency transfer assessments to date, demonstrating fractional frequency uncertainties below 10^–19^ over >700 km long fibre links^27,28,30^. However, our approach based on remote ultrastable laser comparison has two distinct advantages over loop-back schemes. First, it directly reveals the noise of one individual link A-B, rather than the combined link A-B-A. This is important where the two fibres involved differ significantly in terms of loss, scattering and parasitic reflections, effects that can impair noise cancellation schemes, or where the correlation of noise between the two fibres is unclear, for example because they take different paths. The former is the case of the NPL-LPL fibre link, where one of the fibres suffers from excessive reflections (probably caused by connectors beyond our access). In such a case, frequency noise on the fibre link A-B may be masked by the potentially higher noise of the fibre link B-A. Second, many applications of fibre links involve short segments of fibre, or entire systems such as frequency combs, that cannot easily be looped back over, and whose noise contribution cannot be assessed because of that. It is usually assumed to be negligible, based on the comparatively short length of fibre. In the fibre network between NPL and PTB, the four long stabilised segments are interconnected by few-metre, single-fibre two-way links at LPL and UoS and a common 1,542 nm transfer laser at LNE-SYRTE (Fig. 1), whose noise is common-mode rejected. Loop-back diagnostic data are available for the four long stabilised segments, but not for the short two-way links where we rely on estimates and reasonable assumptions to justify neglecting their contribution. The remote ultrastable laser comparison offers a monitoring tool capable of directly validating the operation of these interface sites based on observed instabilities. The connection to the frequency combs and the corresponding beat signal processing is also included in the remote ultrastable laser comparison, yielding a test across the full measurement chain.

**Fig. 3:**
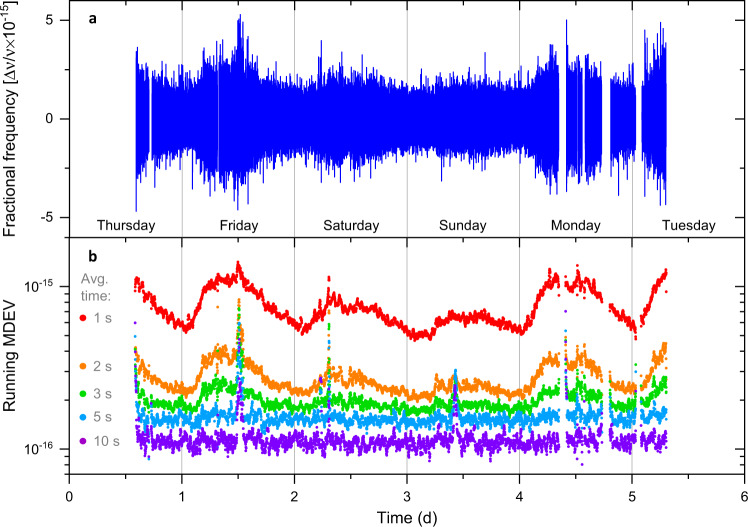
Ultrastable laser comparison as a means of extracting the total fibre link noise as a function of time. **a** Frequency comparison of distant ultrastable lasers (data taken in July 2019). A high-pass filter with a cut off frequency of 1 mHz is used to decouple the short-term instability of the optical fibre link from the drift of the ultrastable lasers (with a characteristic time >1000 s). **b** The total phase-stabilised 2220 km fibre link residual noise as a running instability of the high-pass filtered comparison of ultrastable lasers, calculated using data sets of duration 1000 s for 1 s to 10 s averaging time. The variations in environmental noise picked up along the fibre over six consecutive days of continuous operation can be resolved for 1 s averaging time. The days of the week are shown to highlight the correlation with human activity. Comparison noise averages down to $1\times {10}^{-16}$ instability at 10 s integration time. The spikes in instability are glitches in the link stabilisation.

The 1 s frequency instability (MDEV) of the remote ultrastable laser comparison, plotted as a function of time (Fig. 3), displays environmental noise picked up along the fibre links, which varies from $6\times {10}^{-16}$ during the night to $1.2\times {10}^{-15}$ during working days, and $8\times {10}^{-16}$ and $7\times {10}^{-16}$ on Saturday and Sunday, respectively. The measurements shown in Fig. 2 were taken during the night. In Fig. 3 the data are high-pass filtered with a cut-off frequency of 1 mHz to decouple link noise from ultrastable laser behaviour with characteristic time above 1000 s. This method is used to extract the total link noise from 1 s to 10 s averaging time. We measure that the environmental noise averages down to $1\times {10}^{-16}$ at 10 s, establishing a reference value down to which the total fibre link instability can be monitored using this approach. For averaging times beyond 10 s the behaviour of the link and ultrastable lasers can no longer be easily decoupled. Further improvements in ultrastable lasers would extend the characterisation of the fibre link noise to longer averaging times.

### Investigation on dissemination of ultrastable light to distant users

These results over the 2220 km long European fibre network give us the opportunity to investigate the dissemination of ultrastable light to distant users. As an example, we consider how a generic user operating an optical clock can benefit from receiving light from a distant laser hundreds of kilometres away through an optical fibre. The stability of the remote ultrastable laser enables significantly improved atomic excitation probability, an increased interrogation time, and therefore a better stability performance of the local optical clock, while the link noise at short timescales can be suppressed by the same medium-stability laser that is already part of the local clock laser system. To quantify this scenario, we approximate the measured phase noise power spectral density (PSD) ${S}_{\varphi }({{{f}}})$ of the ultrastable lasers and link ensemble to the simplified noise model

2 $${S}_{\varphi }(f)={\nu }_{0}^{2}\mathop{\sum }\limits_{m=-2}^{2}{h}_{m}{f}^{m-2}$$

with *f* the Fourier frequency of the phase noise spectrum, ν_0_ = 194 THz the carrier frequency of the optical link and *h*_*m*_ parameters for the fundamental noise types (*h*_−2_ random frequency walk, *h*_−1_ flicker frequency, *h*_0_ white frequency, *h*_1_ flicker phase and *h*_2_ white phase). The terms *h*_−1_ and *h*_−2_ are attributed to the ultrastable laser since a reference optical cavity at the fundamental thermal noise floor displays flicker frequency noise, and the residual long term fluctuations (after removing the linear drift) of the cavity can be described as random frequency walk. The independent local measurements show that the individual ultrastable lasers can support instability below $1\times {10}^{-16}$ at 1 s. The values found for *h*_0_, *h*_1_ and *h*_2_ in the fibre link comparison all correspond to significantly larger instabilities and are therefore attributed to the fibre link. At high frequencies, white phase noise is the leading contributor. The *h*_*m*_ values are computed from the comparison of the ultrastable lasers through the $2220\,{{{{{\rm{km}}}}}}$ long link network (Table 1). For simplicity the fibre link is approximated as three concatenated and individually stabilised segments of equal length of about 740 km. The shorter 45 km long link as well as the few-metre single-fibre connections in between (Fig. 1) are neglected. This enables us to estimate the delay-unsuppressed phase noise coefficient^36^ *h*_L_ of the fibre link network considered here, using the expression

3 $${h}_{{{\rm{L}}}}=\frac{\left(\frac{{h}_{2}}{3}\right){\nu }_{0}^{2}}{a{\left(\frac{2\pi n}{c}\right)}^{2}{\left(\frac{{L}_{{{{{{\rm{link}}}}}}}}{3}\right)}^{3}},$$

where ${L}_{{{{{{\rm{link}}}}}}}\approx 2220\,{{{{{\rm{km}}}}}}$ is the full length of the link, $a\approx 1/3$ in the approximation of uniformly distributed noise, $n\approx 1.4$ is the effective group index of refraction of the fibre, *c* is the speed of light and the division by three of *h*_2_ and ${L}_{{{{{{\rm{link}}}}}}}$ accounts for approximating the link as three equal segments. We find that for the European fibre link network the delay-unsuppressed phase noise coefficient is ${h}_{{{{{{\rm{L}}}}}}}\cong 0.5\,{{{{{{\rm{Hz}}}}}}}^{2}{{{{{{\rm{Hz}}}}}}}^{-1}{{{{{{\rm{km}}}}}}}^{-1}$, comparable to what is reported in ref. ^25^ and about 140 times lower than what is reported in ref. ^14^. The work described therein has been carried out in a metropolitan environment where a higher level of noise is expected. The fibre link discussed here covers both metropolitan and rural areas.

**Table 1:** Fundamental noise types budget and simplified noise model parameters.

Type of noise (attributed source)	Noise estimated from the comparison of ultrastable lasers through the 2220 km long fibre link network	Model for the composite phase noise PSD of the fibre-distributed ultra-stable laser with a local clean-up laser, used to calculate the optical lattice clock Dick instability for the 500 km 3-segment link	Model to evaluate the excitation probability of an optical clock and QPN of an ion optical clock for light distributed over the 500 km 3-segment link
White phase *(optical fibre link)*	${h}_{2}=4{.8\times 10}^{-30}\,{{{{{{\rm{Hz}}}}}}}^{-3}$	$${h}_{2}^{\ast }=\Bigg\{\begin{array}{ll}{h}_{2}\times {\left(\frac{500\,{{{{{\rm{km}}}}}}}{2220\,{{{{{\rm{km}}}}}}}\right)}^{3} & {{\mbox{for}}}\;\;f < 7\,{{\mbox{Hz}}}\hfill\\ 0\hfill & {{\mbox{for}}}\;\;f\ge 7\,{{\mbox{Hz}}}\end{array}$$	${h}_{2}^{\ast }$
Flicker phase *(optical fibre link)*	${h}_{1}=1.8\times {10}^{-30}\,{{{{{{\rm{Hz}}}}}}}^{-2}$	$${h}_{1}^{\ast }=\Bigg\{\begin{array}{ll}{h}_{1}\times {\left(\frac{500\,{{{{{\rm{km}}}}}}}{2220\,{{{{{\rm{km}}}}}}}\right)}^{3} & {{\mbox{for}}}\;\;f < 7\,{{\mbox{Hz}}}\hfill\\ 0\hfill & {{\mbox{for}}}\;\;f\ge 7\,{{\mbox{Hz}}}\end{array}$$	0
White frequency *(optical fibre link)*	${h}_{0}=1.9\times {10}^{-31}{{{{{{\rm{Hz}}}}}}}^{-1}$	$${h}_{0}^{\ast }=\Bigg\{\begin{array}{ll}{h}_{0}\times {\left(\frac{500\,{{{{{\rm{km}}}}}}}{2220\,{{{{{\rm{km}}}}}}}\right)}^{3} & {{\mbox{for}}}\;\;f < 7\,{{\mbox{Hz}}}\hfill\\ 0\hfill & {{\mbox{for}}}\;\;f\ge 7\,{{\mbox{Hz}}}\end{array}$$	0
Flicker frequency *(ultrastable lasers)*	${h}_{-1}=3.6\times {10}^{-33}$	$${h}_{-1}^{\ast }=\Bigg\{\begin{array}{ll}\frac{{(5\times {10}^{-17})}^{2}}{2\,{{{{\mathrm{ln}}}}}(2)} & {{\mbox{for}}}\;\;f < 7\,{{\mbox{Hz}}}\hfill\\ \frac{{(5\times {10}^{-15})}^{2}}{2\,{{{{\mathrm{ln}}}}}(2)} & {{\mbox{for}}}\;\;f\ge 7\,{{\mbox{Hz}}}\end{array}$$	${h}_{-1}^{\ast }$
Random walk *(ultrastable lasers)*	${h}_{-2}=2.2\times {10}^{-36}\,{{{{{\rm{Hz}}}}}}$	${h}_{-2}^{\ast }={h}_{-2}$	0

The noise measured in the comparison of ultrastable lasers is expressed in terms of fundamental noise types (*h*_*m*_). We derive a simplified noise model (${h}_{m}^{\ast }$) obtained by referencing a local medium-stability laser at $5\times {10}^{-15}$ to a distant ultrastable laser at $5\times {10}^{-17}$ delivered through a 500 km 3-segment link, under the assumption of dominant delay-unsuppressed link noise^36^, following ${\sigma }_{{{{{{\rm{link}}}}}}}^{2}\propto {L}_{{{{{{\rm{link}}}}}}}^{3}$, with ${\sigma }_{{{{{{\rm{link}}}}}}}$ and ${L}_{{{{{{\rm{link}}}}}}}$ the residual instability and length of the link, respectively. The 500 km 3-segment link is equivalent to a 240 km 1-segment link in the approximation of delay-unsuppressed and uncorrelated segment link noise. The parameters for the shorter link are used to evaluate the impact of referencing a local laser to a distant ultrastable laser in terms of reduction of the Dick noise instability in a local optical clock. For simplicity, only the dominant link white phase noise and laser flicker frequency noise parameters are considered to evaluate the improvement in excitation probability and QPN instability of a local optical clock.

**Fig. 4:**
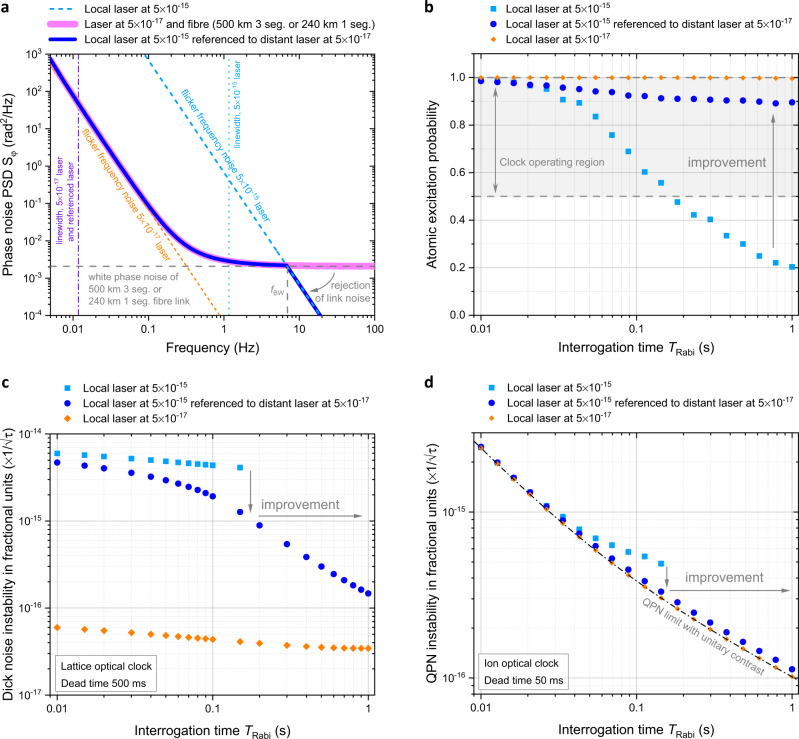
Estimated effect of fibre-disseminated ultrastable light on atomic excitation probability and instability of remote optical clocks. **a** Modelled phase noise for a medium-stability laser at the user’s location (cyan dashed line) and for a distant ultrastable laser, whose light is disseminated to the user through a hypothetical 500 km 3-segment link or 240 km 1-segment link (pink solid line). Medium-stability laser referenced to the distant ultrastable laser (blue solid line). **b** Simulated excitation probability of a hypothetical optical clock at the user’s location as a function of the Rabi interrogation time ${T}_{{{{{{\rm{Rabi}}}}}}}$ obtained using a local medium-stability laser (cyan squares), a medium-stability laser referenced to a distant ultrastable laser (blue circles) or a local ultrastable laser (orange diamonds). The error bars (see the Methods section) are within the size of the markers. **c** Instability of a hypothetical lattice clock (Sr lattice with clock transition at 698 nm) with 500 ms dead-time as a function of ${T}_{{{{{{\rm{Rabi}}}}}}}$ obtained using a medium-stability laser (cyan squares), a medium-stability laser referenced to a distant ultrastable laser (blue circles) or a local ultrastable laser (orange diamonds). **d** Instability of a hypothetical ion optical clock (Yb ion with clock transition at 467 nm) with a dead-time of 50 ms as a function of ${T}_{{{{{{\rm{Rabi}}}}}}}$ using a local medium-stability laser (cyan squares), a medium-stability laser referenced to a distant ultrastable laser (blue circles) or a local ultrastable laser (orange diamonds). The error bars (see the “Methods” section) are within the size of the markers. In (**c**) and (**d**), points corresponding to an excitation probability <0.5 have been omitted as in practice an optical clock would not be able to reliably operate below this excitation value.

Building on a previous demonstration in ref. ^14^ we revisit the approach of using a local clean-up oscillator to limit the amount of white phase noise seen at the end of a fibre link and to transfer stability from a distant laser, in the context of a link with a length of hundreds of kilometres and with a distant ultrastable laser with state-of-the-art instability at $5\times {10}^{-17}$. For the local clean-up oscillator we consider a medium-stability laser with a flicker frequency noise floor at $5\times {10}^{-15}$ (about 1 Hz linewidth), as this performance is accessible with a relatively simple experimental configuration and nowadays commercially available. Assuming the fundamental limitation of delay-unsuppressed noise for the link we use the scaling law^36^ ${\sigma }_{{{{{{\rm{link}}}}}}}^{2}\propto {L}_{{{{{{\rm{link}}}}}}}^{3}$, with ${\sigma }_{{{{{{\rm{link}}}}}}}$ and ${L}_{{{{{{\rm{link}}}}}}}$ the residual instability and length of the link, respectively, to study the hypothetical scenario of a shorter 500 km 3-segment link (Table 1). This 500 km 3-segment link is, in terms of residual instability, equivalent to a 240 km 1-segment link, assuming delay-unsuppressed and uncorrelated segment link noise, as ${(\frac{500\,{{{{{\rm{km}}}}}}}{2220\,{{{{{\rm{km}}}}}}})}^{3}\cong \frac{1}{3}{(\frac{240\,{{{{{\rm{km}}}}}}}{2220/3\,{{{{{\rm{km}}}}}}})}^{3}$. We consider this scenario as a compromise between distance and noise level, providing a useful reference while not over restricting the location of the ultrastable laser. The high level of delay-unsuppressed noise of the full 2220 km 3-segment link does not allow high contrast excitation of an atomic clock transition even with a local clean-up oscillator. A 500 km 3-segment link (or 240 km 1-segment) in terms of noise is equivalent to a 2220 km link with ${N}_{{{{{{\rm{seg}}}}}}}\approx 28$ segments, as $1\cong \frac{1}{{N}_{{{{{{\rm{seg}}}}}}}}{(\frac{240\,{{{{{\rm{km}}}}}}}{2220/{N}_{{{{{{\rm{seg}}}}}}}\,{{{{{\rm{km}}}}}}})}^{3}$, each one with a length of about 80 km. The choice of this 500 km 3-segment link (or 240 km 1-segment) scenario will be further put in perspective later in the text. The clean-up laser can be referenced to the output of the stabilised fibre link using a feedback or feedforward scheme. We assume a transfer bandwidth *f*_BW_ such that we can reject link white phase noise for $f > {f}_{{{{{{\rm{BW}}}}}}}$ and realise a composite phase noise PSD dominated by the distant ultrastable laser at low frequencies and by the local laser at high frequencies (Table 1 and Fig. 4a). The value of the transfer bandwidth *f*_BW_ is defined by the intersection of the link white phase noise and local clean-up laser flicker frequency noise, corresponding to the value ${f}_{{{{{{\rm{BW}}}}}}}\approx ({h}_{-1}^{\ast }/{h}_{2}^{\ast })^{1/3}\approx 7\,{{{{{\rm{Hz}}}}}}$, where ${h}_{2}^{\ast }={h}_{2}\times {(\frac{500\,{{{{{\rm{km}}}}}}}{2220\,{{{{{\rm{km}}}}}}})}^{3}$ is the link white phase noise scaled for a 500 km 3-segment link, or equivalently for a 240 km 1-segment link, and ${h}_{-1}^{\ast }={({5\times 10}^{-15})}^{2}/{{{{{\rm{2ln}}}}}}(2)$ is the flicker frequency noise of the local clean-up laser.

With this simplified model we can evaluate the potential benefits of referencing the local medium-stability laser to the distant ultrastable laser in terms of excitation probability (Fig. 4b) and fractional frequency instability of an hypothetical optical clock at the user’s location (Fig. 4c, d). We predict that the excitation probability of a generic optical clock can be significantly improved to maintain a level of 0.9 for the range of Rabi interrogation time ${T}_{{{{{{\rm{Rabi}}}}}}}$ considered here from 0.1 s to 1 s. Having high atomic excitation probability is key to reliably operate an atomic clock in measurement mode, where the interrogating light is corrected experimental cycle after cycle to be stabilised on the atomic transition by the atomic servo. We define here a threshold level of 0.5 for the atomic excitation probability, and below this value we consider the atomic clock non-operable in practice and we do not report the associated frequency stability performance. For an optical lattice clock (we have considered the case of a Sr lattice clock with ${\nu }_{{{{{{\rm{clock}}}}}}}=429\,{{{{{\rm{THz}}}}}}$) with a fixed preparation time of 500 ms (Fig. 4c) we predict that the clean-up oscillator scheme significantly extends the possible Rabi interrogation time ${T}_{{{{{{\rm{Rabi}}}}}}}$ from 150 ms to beyond 1 s leading to a Dick noise limited instability^37,38^ performance of $1.5\times {10}^{-16}/\sqrt{\tau }$ ($\tau$ averaging time in seconds) at ${T}_{{{{{{\rm{Rabi}}}}}}}=1\,{{{{{\rm{s}}}}}}$, more than 25 times more stable than what is possible with a local laser at a $5\times {10}^{-15}$ and only about a factor of 5 less stable than what would be achievable with a local $5\times {10}^{-17}$ ultrastable laser. We also note that the effectiveness of the clean-up oscillator scheme, in improving the Dick noise limited stability, increases with the Rabi interrogation time, this is because the Dick effect acts as a low pass filter with the frequency corner at $1/{T}_{{{{{{\rm{Rabi}}}}}}}$ on the link noise. This observation is important as it makes the advent of more stable lasers beneficial to the effectiveness of the clean-up oscillator scheme in two concurrent ways: (1) as just discussed it increases ${T}_{{{{{{\rm{Rabi}}}}}}}$, reducing the atomic sensitivity to link noise, (2) it widens the separation between the frequency where the link noise crosses over with the noise from the distant laser and the frequency where it crosses over with the noise of the clean-up laser (Fig. 4a), therefore simplifying the technical implementation of the clean-up oscillator scheme. For a single ion optical clock (we have considered the case of the Yb^+^ with ${\nu }_{{{{{{\rm{clock}}}}}}}=642\,{{{{{\rm{THz}}}}}}$) with a fixed preparation time of 50 ms (Fig. 4d) the model shows that with the clean-up oscillator scheme it is possible to extend the Rabi interrogation from 150 ms to beyond 1 s resulting in a quantum projection noise (QPN) limited instability of $1.1\times {10}^{-16}/\sqrt{\tau }$ at ${T}_{{{{{{\rm{Rabi}}}}}}}=1\,{{{{{\rm{s}}}}}}$, about 5 times more stable than what is possible with a local laser at a $5\times {10}^{-15}$ and basically at the same level of what would be achievable with a local $5\times {10}^{-17}$ultrastable laser.

The calculations have so far been based on the observed night-time performance of the 2220 km link. Repeating the simulations for operation during the day, we find that the optical clock excitation probability (as well as effective QPN limited stability) is reduced by a factor of 1.1, and Dick noise instability is increased by a factor of 1.5. Using a definition of laser linewidth $\Delta {\nu }_{{{{{{\rm{laser}}}}}}}$ via

4 $$\int_{\Delta {\nu }_{{{{{{\rm{laser}}}}}}}/2}^{\infty }{S}_{\varphi }({{{f}}})={1\,{{{{{\rm{rad}}}}}}}^{2}\,$$

we estimate a FWHM linewidth $\Delta {\nu }_{{{{{{\rm{laser}}}}}}}$ for the medium stability laser of about 1 Hz. When referenced to the distant ultrastable laser, the linewidth approaches that of a $5\times {10}^{-17}$ instability laser at 10 mHz (Fig. 4a).

**Fig. 5:**
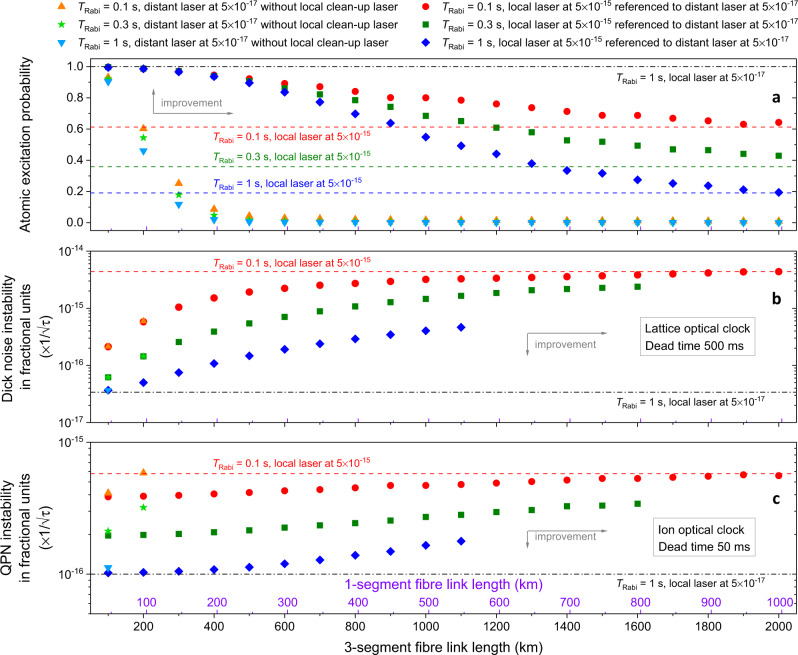
Estimated atomic excitation probability and instability of an optical clock receiving light from a distant ultrastable laser as a function of the optical fibre length. **a** Simulated excitation probability of a hypothetical optical clock at the user’s location as a function of the fibre link length, with and without a local clean-up laser, for a Rabi interrogation time ${T}_{{{{{{\rm{Rabi}}}}}}}$ of 0.1 s (red circles and orange upward triangles), 0.3 s (dark green squares and green stars) and 1 s (blue diamonds and cyan downward triangles). The error bars (see the “Methods” section) are within the size of the markers. **b** Instability of a hypothetical optical lattice clock (Sr lattice with clock transition at 698 nm) with 500 ms dead-time, with and without a local clean-up laser, for a Rabi interrogation time ${T}_{{{{{{\rm{Rabi}}}}}}}$ of 0.1 s (red circles and orange upward triangles), 0.3 s (dark green squares and green stars) and 1 s (blue diamonds and cyan downward triangles). **c** Instability of a hypothetical ion optical clock (Yb ion with clock transition at 467 nm) with a dead-time of 50 ms as a function of the fibre link length, with and without a local clean-up laser, for a Rabi interrogation time ${T}_{{{{{{\rm{Rabi}}}}}}}$ of 0.1 s (red circles and orange upward triangles), 0.3 s (dark green squares and green stars) and 1 s (blue diamonds and cyan downward triangles). The error bars (see the “Methods” section) are within the size of the markers. In (**b**) and (**c**), points corresponding to an excitation probability <0.5 have been omitted as in practice an optical clock would not be able to reliably operate below this excitation value.

Our simplified model does not consider the detail of the spatial distribution of the noise sources along the fibre link, assuming a homogeneous distribution that is derived from a direct measurement of a long fibre link under real operating conditions. The distance of 500 km 3-segment link is chosen as trade-off between stability enhancement of the user’s laser and geographical range of application. In Fig. 5 we study how the atomic excitation probability and the instability of typical optical clocks depend on the length of the fibre link considered (both 3-segment and 1-segment), with and without a local clean-up laser, and for different Rabi interrogation times. The case without local clean-up laser is derived by neglecting for simplicity the fibre link servo spike^36^ at ${f}_{{{{{{\rm{servo}}}}}}}\approx 1/(4\tau )$, where $\tau =n{L}_{{{{{{\rm{link}}}}}}}/c$ is the one-way propagation time along the link, and by considering a flicker frequency roll-off for frequencies above ${f}_{{{{{{\rm{servo}}}}}}}$. We make this assumption as the amplitude of the link servo spike depends on the technical implementation of the link and, here, we would like to derive a general baseline to compare with. In the practical implementation of the clean-up oscillator scheme the link servo spike would be filtered out by the local clean-up laser as ${f}_{{{{{{\rm{BW}}}}}}}\ll {f}_{{{{{{\rm{servo}}}}}}}$. Even neglecting the contribution of the link servo spike and therefore underestimating the link noise, the model predicts (see Fig. 5a) a fast degradation of the atomic excitation probability without local clean-up laser, reaching the threshold of non-operability for an optical clock (set at 0.5 contrast) at about 200 km 3-segment (100 km 1-segment) link length. With the local clean-up laser, the non-operability threshold is reached at about 1100 km 3-segment (550 km 1-segment) for ${T}_{{{{{{\rm{Rabi}}}}}}}=1\,{{{{{\rm{s}}}}}}$ and at about 1600 km 3-segment (800 km 1-segment) for ${T}_{{{{{{\rm{Rabi}}}}}}}=300\,{{{{{\rm{m}}}}}}s$. For a very long link length, the atomic excitation asymptotically approaches the value corresponding to the local clean-up laser. For a 2000 km 3-segment link, similar to the one considered in this work, there is no improvement in atomic excitation probability in referencing a local clean-up laser of currently available specifications to a distant ultrastable laser as the link noise is simply too high. The frequency instability of both an ion and a lattice optical clock (Fig. 5b,c) would therefore benefit from a distant ultrastable laser at $5\times {10}^{-17}$ up to a link distance of 1100 km 3-segment (550 km 1-segment) for ${T}_{{{{{{\rm{Rabi}}}}}}}=1\,{{{{{\rm{s}}}}}}$. At this link distance and interrogation time, a lattice optical clock (Fig. 5b) would improve its stability by about one order of magnitude with respect to what is achievable with a local laser at $5\times {10}^{-15}$, and would be about one order of magnitude less stable than with a local ultrastable laser at $5\times {10}^{-17}$. At the same distance and interrogation time an ion optical clock (Fig. 5c) would improve its stability by about a factor of 3, with respect to what achievable with a local laser at $5\times {10}^{-15}$, and would be less than a factor of 2 less stable than with a local ultrastable laser at $5\times {10}^{-17}$. The effectiveness of the clean-up oscillator scheme increases for longer interrogation time, as discussed earlier, and it decreases for larger link length as more link noise is injected. The link length of 500 km 3-segment (240 km 1-segment) chosen for the analysis in Fig. 4 enables high atomic excitation probability at all the relevant interrogation times considered here, while there would be no significant atomic excitation probability without clean-up laser at this link length.

This dissemination scheme could be scaled up geographically by further segmenting the link or by using ultrastable lasers at the $5\times {10}^{-17}$ level to regenerate stable light every about one thousand kilometres in the fibre network, depending on the level of noise in the environment surrounding the fibre. Shorter distances between ultrastable lasers will be required for instance in metropolitan areas, and longer distances will be possible in rural locations and seafloor implementations^39^.

## Discussion

We have demonstrated a comparison of two geographically distant state-of-the-art ultrastable lasers through the longest ever reported optical fibre link network. We have measured the total fibre link short-term instability, without having to loop back light through a second link, as typically realised to characterise link performance. We have assessed the potential for a clean-up oscillator scheme to broadcast ultrastable light to distant users through optical fibres. This scheme can bring the instability performance of remote optical clocks close to that achievable with direct access to ultrastable lasers and it can lead to a significant simplification of the local lasers, contributing to a variety of optical clock uses from fundamental research^8–13^, to applications such as precise timekeeping^1–7^ and relativistic geodesy^14–16^. We have highlighted how future improvements in ultrastable lasers can bring several concurrent advantages. First, they would extend the stability characterisation of the fibre link noise, without loop-back light through a second link, to longer averaging times. Second, they would increase the range of accessible ${T}_{{{{{{\rm{Rabi}}}}}}}$, reducing the atomic sensitivity to link noise via the Dick effect. Finally, more stable lasers would simplify the technical implementation of the clean-up oscillator scheme as the relevant cross over frequencies would be more separated. Disseminating stable light from a common ultrastable laser to multiple users opens the possibility to contribute to the implementation over larger geographical distances of more advanced interrogation techniques involving compound clocks, which – with proper synchronization^5–7,14^, zero-dead-time operation^2^, dynamical decoupling protocol^40^, quantum nondemolition measurement^41^ or differential spectroscopy^42^ – can overcome the limit imposed by the finite coherence time of the ultrastable laser. Dissemination of ultrastable light has broad impact, with applications including characterisation of distant laser sources, precision spectroscopy with improved time and frequency distribution^43–46^ and seismology through optical fibres^47^. Recent demonstrations of high fidelity transfer of the properties of metrological light from the optical domain to the microwave^48,49^ and radio frequency^50,51^ domain, further extend the range of applications of this work to radar^52^, navigation, telecommunication, quantum communication^53^, radio astronomy, synchronisation of radio telescope arrays, very long baseline interferometry (VLBI)^50,51^, geodesy and frequency comparison through VLBI^54,55^, deep-space communication and tracking^56^.

## Methods

### Ultrastable laser at NPL

The room-temperature system at NPL^33^ employs a vibration-insensitive design with a cylindrical ultra low expansion cavity spacer with an outer diameter of 10 cm, mounted with its optical axis in the horizontal plane. We use dielectric mirror coatings on fused silica substrates. We measure a residual fractional frequency sensitivity to acceleration of (6.0, 1.8, 2.4)×10^−11^ m^−1^s^2^ for the vertical, longitudinal, and transverse horizontal directions respectively. These sensitivities were measured at 2 Hz and are about one order of magnitude higher than predicted by finite-element-method simulations, most likely due to inaccuracies in the machining of the spacer. To minimise the frequency instability of the cavity-stabilised laser it was necessary to improve the performance of the commercial active vibration-isolation platform used to support the cavity. This was achieved by adding an additional three-axis seismometer on the floor close to the platform, whose velocity signals, digitally integrated and filtered, were applied to the vibration-isolation system in a feedforward configuration. With this technique we bring the contribution of the vibrations to below $4\times {10}^{-17}$ MDEV fractional frequency instability from 1 s integration time; below the estimated thermal noise floor of $6\times {10}^{-17}$ MDEV. Active stabilisation of the residual amplitude modulation is implemented^57^ to keep its contribution below the thermal noise floor.

### Cryogenic ultrastable laser at PTB

The cryogenic silicon cavity at PTB was developed in a collaboration between PTB and JILA. Details are given elsewhere^18,58^. The reference cavity consists of a 21.2 cm long double cone shaped spacer of single-crystalline silicon with dielectric mirror coatings on silicon substrates. Its optical axis is oriented vertically, and the spacer is held at three points at its symmetry plane to minimise its sensitivity to vibrations ($2.5(1.2),\,0.7(6),\,0.4(5)\times {10}^{-12}\,{{{\rm{m}}}}^{-1}{{{\rm{s}}}}^{2}$). The cavity is operated at the zero thermal expansion temperature of silicon (124 K) in a cryostat that is mounted on a vibration isolation platform. The frequency of a 1542 nm fibre laser is locked to the cavity. In previous comparisons^18^ an instability of the system given by the Brownian thermal noise at an MDEV of $4\times {10}^{-17}$ was observed for averaging times between 1 s and 100 s.

### Sr lattice clock at NPL

The Sr optical lattice clock is operated in a similar configuration as described in the ref. ^59–61^. A sample of $1\times {10}^{3}$ atoms is loaded at a radial (axial) temperature of 2.0 μK (1.1 μK) into a vertical 1D optical lattice trap, at the magic wavelength, with a trap waist of 150 μm and a depth of 42 *E*_r_ (where *E*_*r*_ is the atomic recoil energy), before being optically pumped into the stretched state ^1^*S*_0_ *M*_F_ = ±9/2 in a bias magnetic field sufficient to split the *M*_F_ = +9/2 from the *M*_F_ = -9/2 *π* transitions at 698 nm by 1148 Hz. The atoms are then excited to ^3^*P*_0_ *M*_F_ =  ±9/2 using a 20 ms Rabi *π* pulse on the clock transition, before atoms left behind in the ^1^*S*_0_ manifold are cleared out of the lattice using a 5 ms pulse at 461 nm. Finally, the atoms are interrogated with a Rabi *π* pulse on the clock transition up to a duration of about 1 s, and the excitation fraction is read out on a camera using fluorescence detection. The total dead time in the sequence is 630 ms, mostly consisting of the various stages of laser cooling of Sr.

### Sr lattice clock at PTB

The operation of the Sr lattice clock has been described in ref. ^62,63^. By the application of a cryogenic reference resonator^18^ instead of a long room-temperature one^17,20^, a clock instability at $5\times {10}^{-17}/\sqrt{\tau }$ has now been achieved^64^. A few hundred ^87^Sr atoms are trapped in a nearly horizontally oriented lattice with a waist of 65 µm and a depth of about 80 *E*_*r*_. By optical pumping, the atoms are prepared and interrogated alternately in either stretched spin state *M*_*F*_ = ±9/2. Population remaining in other Zeeman levels is removed by a state purification sequence similar to the one applied in the NPL lattice clock. In addition, the lattice depth is lowered to spill atoms in axial vibrational levels ν_z_ > 1 from the lattice to reduce tunnelling-induced uncertainties. The atoms then are interrogated with an interrogation pulse of duration up to about 1 s. Atomic state preparation and detection require about 650 ms. The cycle time is however often extended by 1 s of dead time to lower power dissipation, thermal gradients, and thus the blackbody-related clock uncertainty. Due to the excellent performance of the ultrastable laser, the clock still achieves an instability below $2\times {10}^{-16}/\sqrt{\tau }$ in this case.

### Frequency comb at NPL

A multi-branched erbium-doped fibre optical frequency comb with a repetition rate of 250 MHz transfers frequency stability from the ultrastable laser at 1064 nm (stabilised on the 48.5 cm optical cavity) to the link laser at 1542 nm and the Sr lattice optical clock laser at 698 nm (pre-stabilised to a $1\times {10}^{-15}$ cavity) via a transfer oscillator technique^65^. The light at these three wavelengths is delivered to the frequency comb through optical fibres, with active path length stabilisation implemented to reject fibre phase noise, with residual transfer instability measured at about $1\times {10}^{-17}$ at 1 s integration time.

### Frequency comb at PTB

Light from the PTB ultrastable silicon cavity stabilised laser at 1542 nm is sent to a single branch of an erbium-doped fibre optical frequency comb, which phase-coherently transfers the stability from the ultrastable laser to the Sr lattice clock interrogation light field at 698 nm, with phase stabilised paths^66^, resulting in residual transfer instability measured at about $8\times {10}^{-18}$ at 1 s integration time. Light at 1542 nm of a laser that is frequency-offset phase-locked to the silicon cavity stabilised laser is sent to the international fibre link network, using a phase stabilisation reference closely matching those at the silicon cavity and at the Sr lattice clock.

### European optical fibre link

Frequency counters at LPL and UoS, referenced and synchronised to GPS receivers, measure the beat-note between light at 1542 nm arriving from NPL, LNE-SYRTE, and PTB. The frequency difference between the PTB and NPL 1542 nm laser light is given by the sum of counted beat frequencies and a fixed offset determined by the path-length stabilisation systems. The frequency combs and counters at NPL and PTB are referenced and synchronised to UTC(NPL) and UTC(PTB)^67^, respectively.

### Frequency evaluation

All frequencies at optical clocks, combs, and links nodes are dead-time-free $\varLambda$-counted with a report interval of 1 s. Drift and fluctuations of the SYRTE laser frequency are common mode due to the synchronous counting across the network and thus have no influence on the measured difference between the PTB and NPL 1542 nm laser light. Data acquired at NPL, LPL, UoS and PTB are time-stamped, converted to an agreed-upon common formalism^68^ and format, and then shared for analysis through a software versioning and revision control system repository (SVN).

### Evaluation of atomic excitation probability and frequency instability with disseminated ultrastable light

To quantify the effect that laser noise has on excitation probability we numerically solve the optical Bloch equations using a simulated local oscillator driving field with a phase noise PSD matching that shown in Table 1 and Fig. 4a. The local oscillator is simulated for a 10 s period with a step size of $100$ μs. The noise associated with referencing a local medium-stability laser to a remote ultrastable laser delivered via a phase stabilised fibre link is modelled as follows.

First, separate time series frequency data are generated representing the local laser noise and the remote laser noise including the additional phase noise introduced by the link (${h}_{m}^{\ast }$ parameters as defined in Table 1). For the noise profile of the hypothetical 500 km 3-segment link (and equivalently 240 km 1-segment), we considered only the dominant term of white phase noise to simplify the simulation. The local and remote noise time series are then high and low pass filtered, respectively, before being summed. We use first order filtering and we have verified that changing the order and type of the filter does not significantly impact the estimation of the excitation probability. Finally, the mean frequency offset over the 10 s period is subtracted.

This combined local oscillator noise time series represents the detuning $\delta (t)$ from the atomic resonance and is used to numerically solve the following set of coupled ordinary differential equations (optical Bloch equations) for a Rabi π-pulse of length ${T}_{{{{{{\rm{Rabi}}}}}}}$ (Rabi frequency $\varOmega$ equal to 1/*T*_Rabi_):

5 $$\frac{d{{{\mathbf{U}}}}(t)}{{{{dt}}}}={{{{{{\mathbf B}} }}}}(t)\times {{{\mathbf{U}}}}(t);\;\;{{{\mathbf{U}}}}(t)=\left[\begin{array}{c}u(t)\\ v(t)\\ w(t)\end{array}\right]\;\;{{{{{\rm{and}}}}}}\;\;{{{{{{\mathbf{B}}} }}}}(t)=\left[\begin{array}{c}-{\varOmega} \\ {0}\\ \delta (t)\end{array}\right]$$

with the initial condition **U**(0) = [0, 0, -1]^T^. The elements of **U**(*t*) are the expectation values of three Pauli matrices, hence the excitation probability is given by $(w(t)+1)/2$. This process is repeated 1000 times and the mean of the final excitation probability is taken to be the excitation probability, the result of which is shown in Fig. 4b, with the error bars (within the size of the markers) given by the standard error of the mean.

The Dick noise displayed in the Fig. 4c, expressed in fractional units, is computed starting from the phase noise PSD ${S}_{\varphi }(f)$ using the expression^37,38^

6 $${\sigma }_{y}^{{{\rm{Dick}}}}(\tau)=\frac{1}{\nu_{{{\rm{clock}}}}}\sqrt{\frac{1}{\tau}\mathop{\sum }\limits_{m=1}^{\infty }\left[{\left(\frac{{g}_{m}^{{{\rm{cos}}}}}{{g}_{0}}\right)}^2+{\left(\frac{{g}_{m}^{{{\rm{sin}}}}}{{g}_{0}}\right)}^2\right]{\left(\frac{m}{{T}_{{{\rm{c}}}}}\right)}^2{S}_{\varphi }\left(\frac{m}{{T}_{{{\rm{c}}}}}\right)}$$

with τ the averaging time, *T*_c_ the clock cycle time and the parameters ${g}_{m}^{{{\rm{cos}}}}$, ${g}_{m}^{{{\rm{sin}}}}$ and *g*_0_ defined by

7 $$\left(\begin{array}{c}{{{{g}}}}_{{{{m}}}}^{{{{{{\rm{cos}}}}}}}\\ {{{{g}}}}_{{{{m}}}}^{{{{{{\rm{sin}}}}}}}\end{array}\right)=\frac{1}{{{{{T}}}}_{{{{{{\rm{c}}}}}}}}{\int }_{0}^{{{{{T}}}}_{{{{{{\rm{c}}}}}}}}{{{g}}}({{{t}}})\left(\begin{array}{c}\cos (2\pi {{{mt}}}/{{{{T}}}}_{{{\rm{c}}}})\\ \sin (2\pi {{{mt}}}/{{{{T}}}}_{{{{{{\rm{c}}}}}}})\end{array}\right){{{dt}}},\;\;{g}_{0}=\frac{1}{{{{{T}}}}_{{{{{{\rm{c}}}}}}}}\int _{0}^{{{{{T}}}}_{{{{{{\rm{c}}}}}}}}{{{g}}}({{{t}}}){{{dt}}}\,$$

with *g*(*t*) the sensitivity function for Rabi spectroscopy^37^

8 $$\,g(t)\approx 0.38\times [\sin ({\varOmega }_{1}(t))(1-\,\cos ({\varOmega }_{2}(t)))+\,\sin ({\varOmega }_{2}(t))(1-\,\cos ({\varOmega }_{1}(t)))]$$

for $0 \; < \; t \; < \; {T}_{{{{{{\rm{Rabi}}}}}}}\,$, and zero otherwise, with

9 $${\varOmega }_{1}(t)\approx 4\frac{t}{{T}_{{{{{{\rm{Rabi}}}}}}}},\;\;{\varOmega }_{2}(t)\approx 4\left(1-\frac{t}{{T}_{{{{{{\rm{Rabi}}}}}}}\,}\right).$$

The effective QPN for an optical single ion clock displayed in Fig. 4d, in fractional units, is computed using the approximated expression^69,70^

10 $${\sigma }_{y}^{{{{{{\rm{QPN}}}}}}}(\tau )\cong \frac{1}{\nu_{{{\rm{clock}}}}}\sqrt{\frac{{p}}{2}\left(1-\frac{{p}}{2}\right)}\frac{0.8}{2\pi {p}{T}_{{{{{{\rm{Rabi}}}}}}}}\sqrt{\frac{{T}_{{{\rm{c}}}}}{\tau }}$$

where *p* is the excitation probability.

## Supplementary information

Supplementary Information

## Data Availability

The data that support the finding of this study are available in the public repository Zenodo with the identifier 10.5281/zenodo.5717954 (ref. ^71^).

## Code availability

The codes used to analyse the data and to produce the plots presented in this work are available from the corresponding authors on request to better provide context and support.
