# Supplementary material for: Comparing ultrastable lasers at 7 × 10−17 fractional frequency instability through a 2220 km optical fibre network
Source: Nat Commun. 2022 Jan 11;13:212. doi: 10.1038/s41467-021-27884-3 (PMC8752831; doi:10.1038/s41467-021-27884-3)
Supplement: Supplementary file 1 — Supplementary Information [file 41467_2021_27884_MOESM1_ESM.pdf]

# Comparing ultrastable lasers at $7\times10^{-17}$ fractional frequency instability through a 2,220 km optical fibre network

## Supplementary Information

We display for completeness in the figures here below the fractional frequency instability of additional datasets of the comparison of the distant ultrastable lasers through the 2,220 km long optical fibre link network.

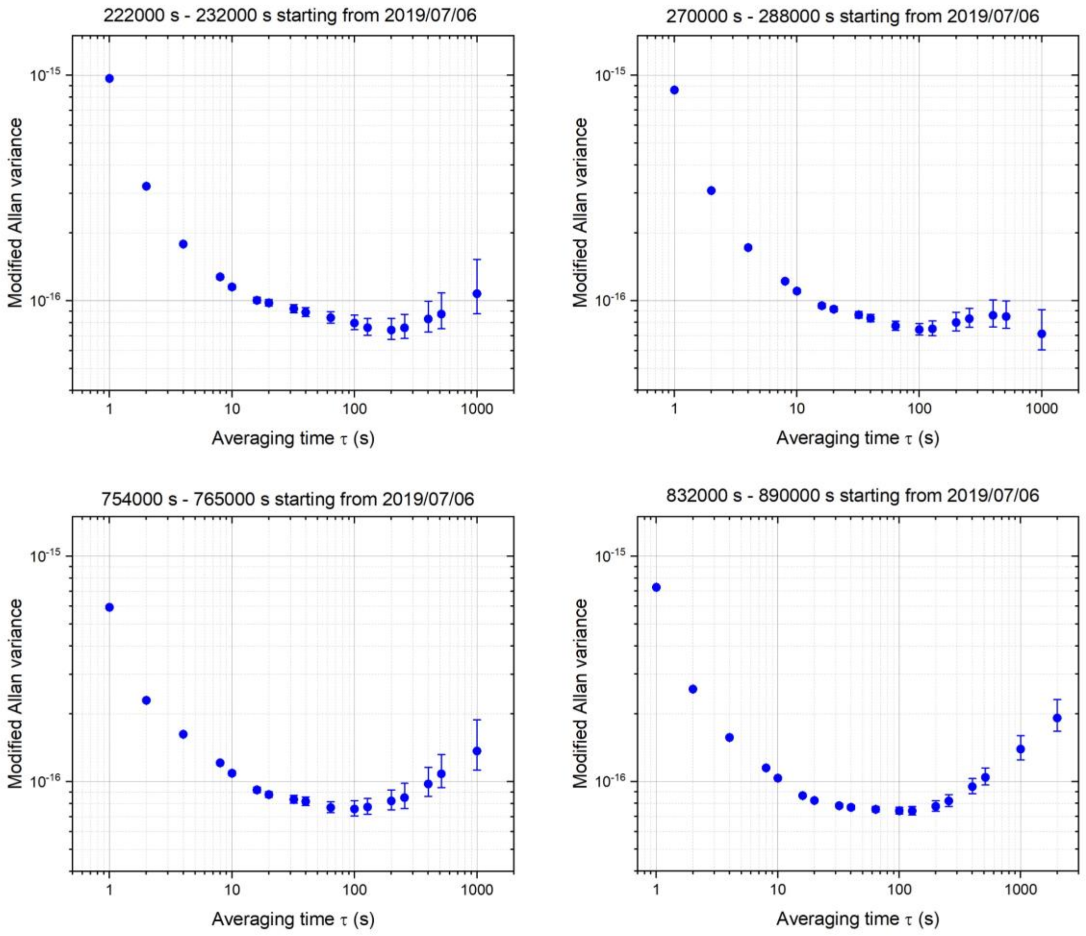


**Supplementary Figure 1 Additional datasets for the experimental results of the comparison of ultrastable lasers through the optical fibre link.** Combined instability measured comparing the NPL room-temperature laser and PTB cryogenic laser (blue circles) through the 2,220 km long European

fibre link network. A linear drift of 40 mHz $s^{-1}$ has been removed in the instability evaluation. The temporal starting point of the seconds displayed above the instability plots is 00:00 UTC of 2019/07/06. The error bars represent the $1\sigma$ uncertainty of the modified Allan deviation.

**Data availability**. The data that support the finding of this study are available in the public repository Zenodo with the identifier DOI: 10.5281/zenodo.5717954 (ref. 71).
